# Diphenyldifluoroketone EF24 Suppresses Pro-inflammatory Interleukin-1 receptor 1 and Toll-like Receptor 4 in lipopolysaccharide-stimulated dendritic cells

**DOI:** 10.1186/s12950-015-0096-x

**Published:** 2015-09-23

**Authors:** Prachi Vilekar, Geeta Rao, Shanjana Awasthi, Vibhudutta Awasthi

**Affiliations:** Department of Pharmaceutical Sciences, College of Pharmacy, University of Oklahoma Health Sciences Center, 1110 North Stonewall Avenue, Oklahoma City, OK 73117 USA

**Keywords:** Inflammation, Dendritic cells, IL-1R, EF24

## Abstract

**Background:**

Unresolved and prolonged inflammation is a pathological basis of many disorders such as cancer and multiple organ failure in shock. Interleukin-1 receptor (IL-1R) superfamily consists of IL-1R1 and pathogen pattern recognition receptor toll-like receptor-4 (TLR4) which, upon ligand binding, initiate pro-inflammatory signaling. The study objective was to investigate the effect of a diphenyldifluoroketone EF24 on the expression of IL-1R1 and TLR4 in lipopolysaccharide (LPS)-stimulated dendritic cells (DCs).

**Methods:**

Immortalized murine bone marrow-derived JAWS II dendritic cells (DC) were challenged with LPS (100 ng/ml) for 4 h. The LPS-stimulated DCs were treated with 10 μM of EF24 for 1 h. The expression levels of IL-1R1 and TLR4 were monitored by RT-PCR, immunoblotting, and confocal microscopy. The effect of EF24 on the viability and cell cycle of DCs was examined by lactate dehydrogenase assay and flow cytometry, respectively.

**Results:**

EF24 treatment suppressed the LPS-induced TLR4 and IL-1R1 expression in DCs. However, the expression levels of IL-1RA and IL-1R2 were not influenced by either LPS or EF24 treatments. These effects of EF24 were associated with a decrease in LPS-induced expression of phospho-NF-kB p65, indicative of its role in the transcriptional control of IL-1R superfamily members. We did not find any significant effect of EF24 on the proliferation or cell cycle of DCs.

**Conclusions:**

The results suggest that EF24 influences IL-1R superfamily signaling pathway in ways that could have salutary effects in inflammation. The pluripotent anti-inflammatory actions of EF24 warrant further investigation of EF24 in inflammatory conditions of systemic nature.

## Background

The restoration of local and systemic homeostasis primarily falls in the jurisdiction of innate arm of immune response, especially in the immediate aftermath of insult. This response manifests itself as inflammation and is meant for the elimination of the cause of injury and for protection from the damage [1]. Unresolved and prolonged inflammation has been identified as a culprit in the pathogenesis of many disorders such as shock, inflammatory bowel diseases and cancer [2]. Mild inflammatory reaction is resolved in normal physiological processing, but moderate-to-severe inflammatory reaction may take a severe toll at both local as well as systemic levels. Inflammation, with or without accompanying sepsis, has also been implicated in multiple organ failure in the victims of hemorrhagic shock and ischemia/reperfusion injury [3–5].

One of the initial steps in inflammatory process involves the recruitment of immune cells, such as neutrophils, monocytes and dendritic cells (DCs). These cells are equipped with cell surface sensors mainly belonging to the interleukin-1 receptor (IL-1R) superfamily. The IL-1R members scout the biological milieu for damage–associated molecular patterns (DAMPs). Upon stimulation, the IL-1R members recruit intracellular adaptor proteins which initiate a molecular cascade culminating into the secretion of effector pro-inflammatory cytokines such as TNF-α and IL-6 [6, 7]. The four major groups of IL-1R superfamily are populated by receptors for IL-1 and IL-18, TLRs, an orphan receptor suppression of tumorigenicity 2 (ST2), and single immunoglobulin IL-1R-related (SIGIRR) [8, 9]. Of these, the most important pro-inflammatory members of IL-1R superfamily are IL-1R1 and toll-like receptors (TLRs). The TLR group is characterized by extracellular leucine-rich repeats, whereas IL-1R1 contains extracellular immunoglobulin (Ig) domains. All the members of IL-1R superfamily have a conserved cytosolic domain called the Toll − IL-1R (TIR) domain, which activates some of the common signaling pathways. The pro-inflammatory effects of IL-1R signaling are primarily mediated by a transcription element nuclear factor-kappa B (NF-kB) [10]. Given the common signaling pathway via NF-kB, IL-1R members are attractive targets for controlling exaggerated inflammation [11–13].

The primary objective of this study was to investigate the effect of EF24, 3,5-bis(2-fluorobenzylidine)-4-piperidone, on the expression of IL-1R members in lipopolysaccharide (LPS)-stimulated DCs. This investigation is an extension of our previous work where we reported the NF-kB inhibitory activity of EF24 in the LPS-stimulated DC model [14]. EF24 is a synthetic chalcone derivative which, because of the presence of α,β-unsaturated carbonyl functionality, participates in nucleophilic addition in biological systems. Such compounds are often referred to as Michael acceptors because they are able to undergo 1,4-additions with thiols. Several synthetic as well as naturally occurring Michael acceptors are known to exhibit anti-inflammatory activity [15, 16]. The inactivation of NF-κB by Michael acceptors is thought to be mediated by their interaction with cysteine residues in either IκB kinase (IKK) or in the DNA-binding domain of NF-κB [17, 18]. EF24 has been show to directly inhibit the catalytic activity of IkappaB kinase (IKK) which is responsible for the phosphorylation of NF-kB inhibitory protein IkappaB [19]. By inhibiting IKKβ, EF24 reduces the phosphorylation-dependent proteasomal degradation of IkappaB which ultimately results in inhibition of activation and nuclear translocation of NF-kB for its transcriptional activity. The results discussed in this report are indicative of the pluripotent anti-inflammatory effects of EF24 involving IL-1R1 and TLR4.

## Methods

The chemical synthesis and characterization of EF24 were performed by the procedures published elsewhere [20]. For the *in vitro* experiments, an aqueous solution of EF24 was prepared in endotoxin-free water (<0.1 EU/ml). A highly-purified lipopolysaccharide (LPS, protein content <0.6 %) of *Escherichia coli* O111: B4 was obtained from Calbiochem (Darmstadt, Germany). The primary rabbit antibodies against mouse antigens were obtained from Cell Signaling Technology (CST, Danvers, MA), Santa Cruz Biotech (SCBT, Dallas, TX), Abcam (Cambridge, MA), and Sigma-Aldrich (Sigma, St. Louis, MO). Horse radish peroxidase (HRP)-conjugated secondary goat anti-rabbit IgG antibody was from CST.

### Cell culture

JAWS II DC cell line is an immortalized and immature DC cell line derived from the bone marrow of C57BL/6 mice (ATCC, Manassas, VA). The cells were maintained in alpha-modified minimum essential medium (Sigma, St Louis, MO) supplemented with 20 % fetal bovine serum (FBS), 4 mM L-glutamine, 100 U/ml penicillin, 100 μg/ml streptomycin, 50 μg/ml gentamicin (Invitrogen, Grand Island, NY) and 5 ng/ml of recombinant murine granulocyte macrophage-colony stimulating factor (GM-CSF; Peprotech, Rocky Hill, NJ). The culture medium was replaced with fresh medium every 48 h. The LPS-stimulated DCs model used in this study has been well-characterized in our previous works [14, 21].

### Drug treatment

The cultured cells were treated with LPS at 100 ng/ml concentration for 4 h, after which 10 μM EF24 was added for 1 h (post-treatment model). The culture medium for drug exposure was kept the same as that described above. In few experiments, the order of LPS and EF24 addition was reversed, i.e. the cells were first treated with EF24 for 1 h, followed by 4 h of LPS treatment (pre-treatment model). Furthermore, in long-term experiments, DCs were stimulated with LPS for 24 h, followed by treatment with EF24 for additional 4 h. The experimental groups included the untreated control DCs, the cells treated with 10 μM EF24 alone, the cells treated with 100 ng/ml LPS alone, and the cells treated with both EF24 and LPS.

### Real-time PCR

The total RNA was extracted from DCs using RNAeasy Mini Kit (Qiagen, Valencia, CA). The purified total RNA was quantified by using absorbance values at 260 nm. Reverse transcriptase reaction was performed for 1 h at 42 °C using 2 μg of total RNA, 1 μg of oligo(dT), 200 U of M-MLV reverse transcriptase enzyme, 500 μM dNTP mix and 25 U of RNAase inhibitor (Promega, Madison, WI). The cDNA was stored at -20 °C till further used. The PCR reaction was performed using SybrGreen II and the Go Taq colorless master mix (Promega, Madison, WI). Briefly, each PCR reaction was set up in triplicate wells in a 96-well plate in a total volume of 25 μl. The reaction mix contained cDNA equivalent to 20 ng of total RNA. The quantitative values of the genes of interest were normalized using β-actin as the endogenous reference, and fold increase over control was calculated using the relative quantification method (2^-ΔΔ^ C_t_ method). The mouse primers (Table 1) were designed and synthesized by either Real Time Primers (RTP, Elkins Park, PA) or Integrated DNA Technologies (Coralville, IA).

**Table 1:** Primers for RT-PCR

Gene	Sequence
TLR1	Forward 5'- TGC AGG AAC TCA ATG TAG CA -3'
	Reverse 5'- TTG ACA AAG TCC CTC AGC TC -3'
TLR2	Forward 5'- GAC GAC TGT ACC CTC AAT GG -3'
	Reverse 5'- TTA AAT GCT GGG AGA ACG AG -3'
TLR4	Forward 5'- AGA CCT CAG CTT CAA TGG TG -3',
	Reverse 5'- GAG ACT GGT CAA GCC AAG AA -3’
TLR5	Forward 5'- GCT CTC CTG CAG ACG TGT AT -3'
	Reverse 5'- AGA TTC CCC GGA ACT TTA TG -3'
TLR6	Forward 5'- TGC AGG AAC TCA ATG TAG CA -3'
	Reverse 5'- TTG ACA AAG TCC CTC AGC TC -3'
IL-1R1	Forward 5'- CGC AGA AGC TGA AGT CTA CG -3',
	Reverse 5'- CAG GTG GCA GAA ATG CTA GA -3'

### Immunoblotting

The whole cell lysates of treated JAWS II DCs were prepared in a lysis buffer (25 mM Tris-HCl, pH 7.4) containing 0.1 % SDS, 1 % Igepal, 2 mM EDTA, 1 mM phenyl methyl sulfonyl fluoride (PMSF), 0.2 mM sodium orthovanadate, 50 mM NaF, 150 mM NaCl and 1 μg/ml each of leupeptin and pepstatin. After protein estimation, about 25 μg of total protein was fractionated on Novex 4-20 % Tris–glycine gradient SDS-PAGE gels (Invitrogen, Carlsbad, CA). The separated proteins were electro-transferred onto nitrocellulose membranes using iBlot gel transfer device (Invitrogen, Carlsbad, CA). The non-specific sites were blocked by incubating the membranes with 5 % skimmed milk in Tris-buffered saline with 0.4 % Tween-20 (TBST). The blocked membranes were incubated overnight at 4 °C with appropriate primary antibodies in TBST. The primary antibodies against TLR4 (Abcam), IL-1R1 (Abcam), IL-1R2 (SCBT), and IL-1RA (SCBT) were used at dilutions recommended by the manufacturers. After probing, the membranes were washed and incubated with HRP-conjugated-anti-rabbit-IgG antibody diluted at 1:5,000 in TBST. The immunoreactive bands were detected by SuperSignal West Femto detection reagent (Thermo Fischer Scientific, Rockford, IL). In order to ensure equal protein loading in the wells, the membranes were stripped using a stripping solution containing 10 % SDS, 0.5 M Tris and *β*-mercaptoethanol (35 μl/ml) at 60 °C for 45 min, and re-probed with anti-actin antibody (Sigma; 1:5,000 in TBST). The blots were imaged and the densitometric readings for the proteins were normalized with those of actin.

### Confocal microscopy

DCs were seeded at a density of 25,000 cells per well in an 8-well chamber slide (Nalge Nunc international, New York, USA). After the treatment with LPS (100 ng/ml) and EF24 (10 μM), the cells were fixed for 20 min with ice-cold 3.5 % paraformaldehyde in PBS. Cell permeabilization was carried out for 20 min on ice with alpha-MEM medium containing 10 % FBS and 0.05 % saponin and 10 mM HEPES [22]. The cells were washed with PBS supplemented with 1 % FBS and 0.05 % saponin (wash buffer). The non-specific binding sites were blocked using PBS containing 10 % normal mouse serum (Sigma, St Louis MO) for 1 h at room temperature. A rabbit polyclonal antibody to mouse TLR4 (Abcam) was added to the cells at a dilution of 1:50 and incubated overnight at 4 °C in a humidified chamber. For NF-kB activation study, we used mouse reactive rabbit monoclonal anti-phospho-NF-kB p65 antibody (CST) at a dilution of 1:200. The cells were washed thrice for 5 min each and incubated with 10 μg/ml Alexa Fluor 488-labeled donkey anti-rabbit IgG antibody (Molecular Probes, Carlsbad, CA) for 1 h. In some experiments, the cells were also labeled with 100 nM rhodamine-phalloidin (Cytoskeleton Inc, Denver CO) for 30 min at room temperature, protected from light. Finally, 1 μg/ml Hoechst 33342 (Molecular Probes, Carlsbad, CA) dye was added to the cells. The confocal microscopic images were obtained at the Oklahoma Medical Research Foundation (Oklahoma City, OK) using the Zeiss LSM-510META laser scanning confocal microscope. The images were acquired with objective lens of 63X as composites or with the x/y stack sizes of 146.2 μm using the band pass filter specifications at 435-485, 560-615 and 505-530.

### IL-1β enzyme-linked immunosorbent assay

Overnight cultures of JAWS II DCs were stimulated with LPS (100 ng/ml) for 4 h followed by 1 h treatment with EF24 (10 μM) without changing the medium. The medium was collected and IL-1β secreted in the medium of cultured JAWS II DCs was measured by an ELISA kit for mouse IL-1β from Biolegend (San Diego, CA). The samples were diluted 1:10 and manufacturer’s recommendations were followed.

### Cell viability and cell cycle analysis

JAWS II DCs were seeded at a density of 15,000 cells per well of a 96-well tissue culture plate and treated with 0.1, 1, 5, 10, 50 μM EF24. After 6, 24, and 48 h of the treatment, both adherent and non-adherent cells were assessed for membrane integrity using CytoScan™ LDH (lactate dehydrogenase) assay kit (GBiosciences, Maryland Heights, MO) and following the kit manufacturer’s instructions. The cell proliferation was examined by hexosaminidase assay [23]. Briefly, the medium was removed and hexosaminidase substrate solution in 7.5 mM citrate buffer (pH 5), p-nitrophenol-*N*-acetyl-beta-D-glucosaminidase (Calbiochem, San Diego, CA) was added at 60 μl per well. The plate was incubated at 37 °C in 100 % humidity for 30 min, before stopping the reaction by adding 90 μl of 50 mM glycine containing 5 mM of EDTA (pH 10.4); absorbance was measured at 405 nm.

To determine the effect of EF24 on cell cycle progression, we treated JAWS II DCs with 1 and 10 μM EF24 for 24 and 48 h. The vehicle-treated cells were used as a negative control. After the treatment periods, both adherent and non-adherent cells were collected and centrifuged at 260 x g for 5 min. The cells were washed with Dulbecco’s phosphate buffered saline (DPBS) (Gibco, Grand Island, NY), fixed in 70 % ice-cold ethanol for 1 h, and stained with a buffer containing 5 μg/ml DNase-free RNase A (Sigma, St Louis, MO), 0.1 % v/v Triton-X 100 and 0.05 mg/ml propidium iodide (Molecular Probes, Carlsbad, CA). The stained cells were incubated at 4 °C for 30 min in dark, before measuring the fluorescence using Becton Dickson FACS Calibur flow cytometer carrying FL2 detector with a band pass filter at 585/45 nm. The percent number of hypodiploid cells undergoing apoptosis (sub-G_1_) and diploid cells (G_0_-G_1,_ S and G_2_-M phases) were determined by analyzing the histogram charts with ModFit software (Verity software house, Topsham, ME).

### Data analysis

Unless otherwise mentioned all the results were analyzed by one-way analysis of variance (ANOVA) applying the Bonferroni post-test using Prism software (GraphPad, San Diego, CA, USA). *p* < 0.05 was considered statistically significant. The RT-PCR results are expressed as fold changes in comparison with control. To determine the mean fluorescence intensity (MFI) values from confocal images, the fluorescence was quantified in at least three different fields of view for a total of more than 25 cells. Wherever applicable, the data from various experiments were presented as mean ± standard error of mean (sem).

## Results

We used immortalized C57BL/6 mouse-derived JAWS II dendritic cells that show typical immunophenotype and morphology when cultured in presence of GM-CSF. The characteristics of these immortalized cells have been described in our previous reports [14, 24].

### EF24 suppresses the LPS-induced TLR4 expression

**Fig. 1:**
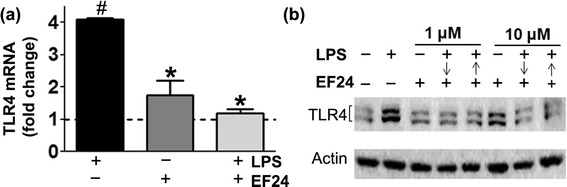
EF24 reduces LPS-induced TLR4 expression in JAWS II DCs. The DCs were treated with 100 ng/ml LPS for 4 h, followed by 1 h treatment with 1 or 10 μM EF24. (**a**) TLR4 mRNA expression (mean ± sem) in DCs treated with EF24. (**b**) TLR4 immunoblot of DCs lysates. The cells were treated with EF24 either after (↓) or before (↑) LPS addition as described in the material and methods. # *p* value < 0.05 vs. control and * *p* value < 0.05 vs. LPS treatment

**Fig. 2:**
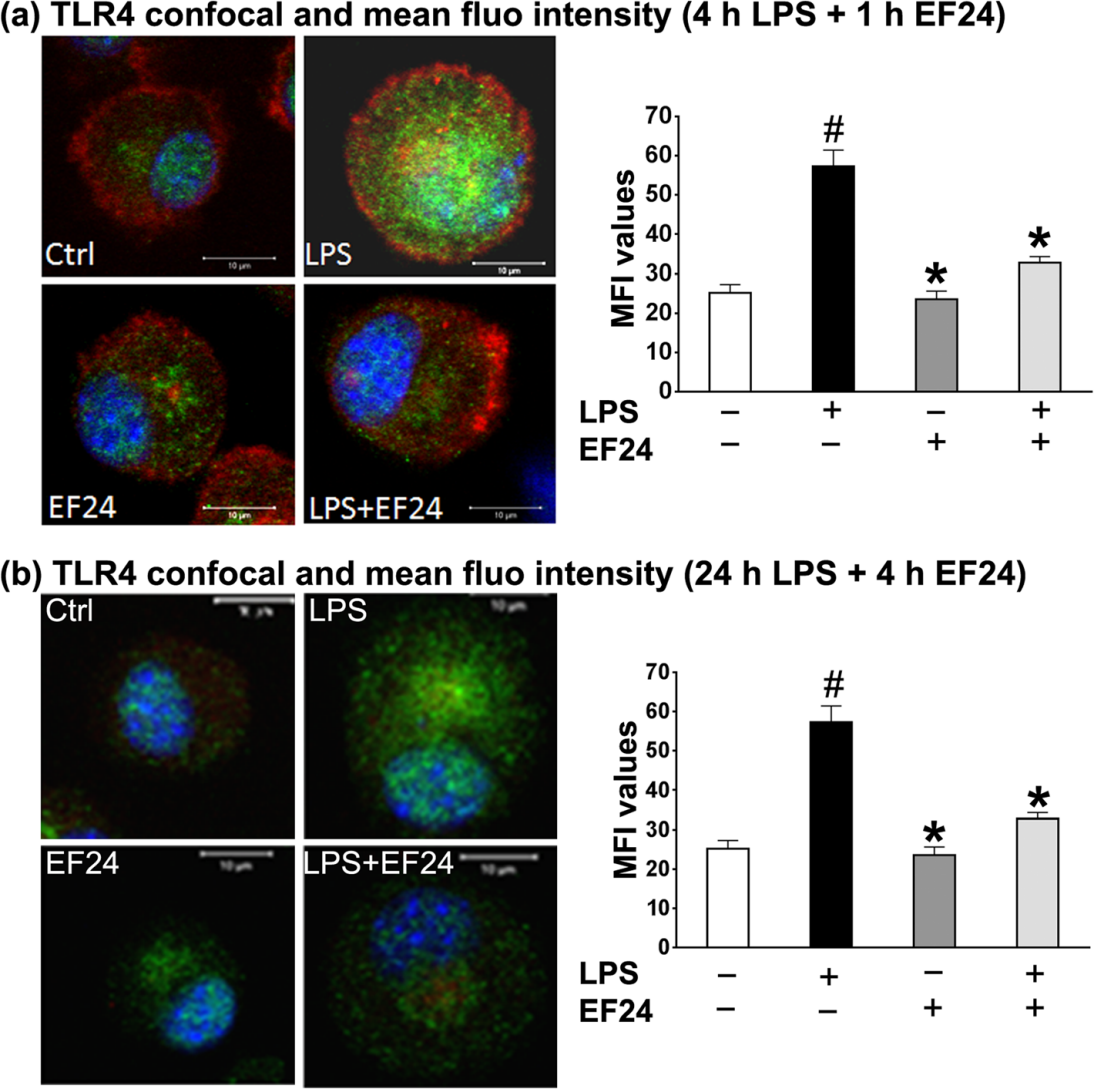
Confocal micrographs and corresponding MFI values showing that EF24 inhibits the LPS-induced TLR4-expression in DCs. The DCs were stimulated for either (**a**) 4 h or (**b**) 24 h, followed by 1 h or 4 h of EF24 treatment, respectively. The cells were stained with Hoechst dye (nucleus, blue) and rhodamine-phalloidin (actin, red) and anti-mouse TLR4 antibody (green). The regions with co-localization of red and green fluorescence appear yellow. The intensity of green fluorescence representing TLR4 (mean ± sem) was quantified in at least three different fields and analyzed by one way analysis of variance and applying the Bonferroni post-test. # *p* value < 0.05 vs. control and * *p* value < 0.05 vs. LPS treatment

We investigated the expression of TLR4 in JAWS II DCs by confocal microscopy, immunoblotting and RT-PCR analyses (Fig. 1). As expected, the exposure of DCs to LPS resulted in a significant increase in expression of TLR4 at both mRNA as well as proteins levels. EF24 significantly (*p* < 0.05) reduced the LPS-induced mRNA levels of TLR4 (Fig. 1a). The immunoblotting for TLR4 showed that both pre- and post-treatment of DCs with EF24 (1 μM as well as 10 μM) reduced the expression of TLR4 protein (Fig. 1b). The confocal microscopy substantiated that EF24 reduced the LPS-induced TLR4 expression (Fig. 2). LPS stimulation increased the MFI values for TLR4 expression by approximately 2.3 fold. Treatment of LPS-stimulated DCs with EF24 showed TLR4 expression that was not significantly different from the control DCs. The suppressive effect of EF24 on the LPS-induced TLR4 expression was also observed when LPS exposure was prolonged to 24 h before treatment with EF24 for additional 4 h (Fig. 2b).

In order to investigate whether LPS and EF24 affect other members of TLR family, we also examined the expression of TLR1, TLR2, TLR5, and TLR6 transcripts in LPS-stimulated DCs. LPS as well as EF24 treatment had no impact on the mRNA levels of these TLRs, showing that the LPS preparation and EF24 solution used in this study were pure and not contaminated with ligands of other pro-inflammatory TLRs (data not shown).

### EF24 reduces the LPS-induced IL-1R1 expression

**Fig. 3:**
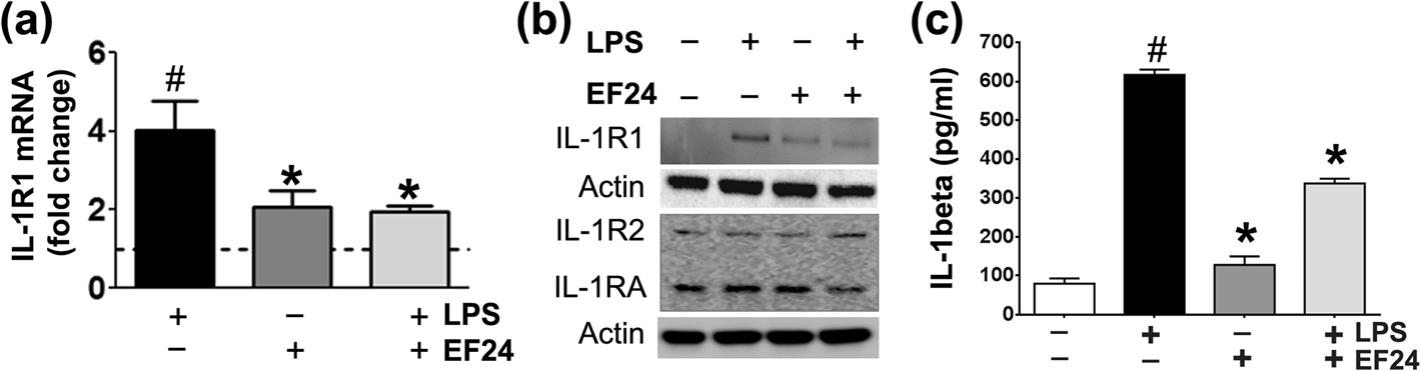
EF24 suppresses the LPS-induced IL-1R1 expression and IL-1β secretion in JAWS II DCs. (**a**) Quantitative-polymerase chain reaction for the measurement of the IL-1R1 mRNA expression (mean ± sem, fold changes as compared to control) in JAWS II DCs that were LPS (100 ng/ml)-stimulated for 4 h, followed by treatment with 10 μM EF24. # *p* value < 0.05 vs. control and * *p* value < 0.05 vs. LPS treatment. (**b**) Immunoblots of IL-1R1, IL-1R2, and IL-1RA expression in JAWS II DCs treated with EF24 and LPS. (**c**) IL-1β secreted in medium of cultured JAWS II DCs. The data is average (± sem) of samples from three separate experiments, each estimated in triplicate (# *p* value < 0.05 vs. control and * *p* value < 0.05 vs. LPS treatment)

IL-1R1 is the primary glycoprotein receptor for pro-inflammatory IL-1α and IL-1β cytokines [9]. We found that LPS treatment of JAWS II DCs significantly increased the expression of IL-1R1. However, after treatment with EF24, the LPS-induced IL-1R1 expression was reduced to basal levels. These effects were observed both at mRNA (Fig. 3a) as well as protein levels (Fig. 3b). EF24 treatment in unstimulated DCS had no effect on the basal expression of IL-1R1. Another receptor for IL-1α and IL-1β is the type 2 IL-1 receptor (IL-1R2). IL-1R2 lacks intracellular signaling domain, rendering it incapable of initiating the IL-1-mediated pro-inflammatory signaling. Therefore, expression of IL-1R2 as a decoy receptor serves as a ‘molecular trap’ for IL-1, with an overall anti-inflammatory impact [25]. Yet another member of IL-1 superfamily is a naturally occurring competitive IL-1 receptor antagonist (IL-1RA). Binding of IL-1RA to IL-1R1 leaves IL-1R1 unable to recruit IL-1R accessory protein for the transduction of IL-1-mediated actions [26]. We investigated the effect of treatment with LPS and EF24 on the expression of IL-1R2 and IL-1RA in DCs (Fig. 3b, lower panel). We found that LPS had no effect on the expression of these two IL-1R1 antagonists. EF24 treatment also did not significantly alter the expression of IL-1R2 and IL-1RA. These results suggest that the anti-inflammatory effects of EF24 in LPS-stimulated DCs are most likely not dependent on its effect on the expression of IL-1R2 and IL-1RA as natural antagonists of IL-1R1 signaling.

We also estimated IL-1β secreted in the medium of LPS-stimulated and EF24-treated DCs (Fig. 3c). LPS significantly increased the medium-bound IL-1β (1.4 × the basal levels) which was reduced by EF24 treatment. EF24 alone had no impact on basal level of IL-1β (Fig. 3c).

### EF24 decreases activation of NF-kB

**Fig. 4:**
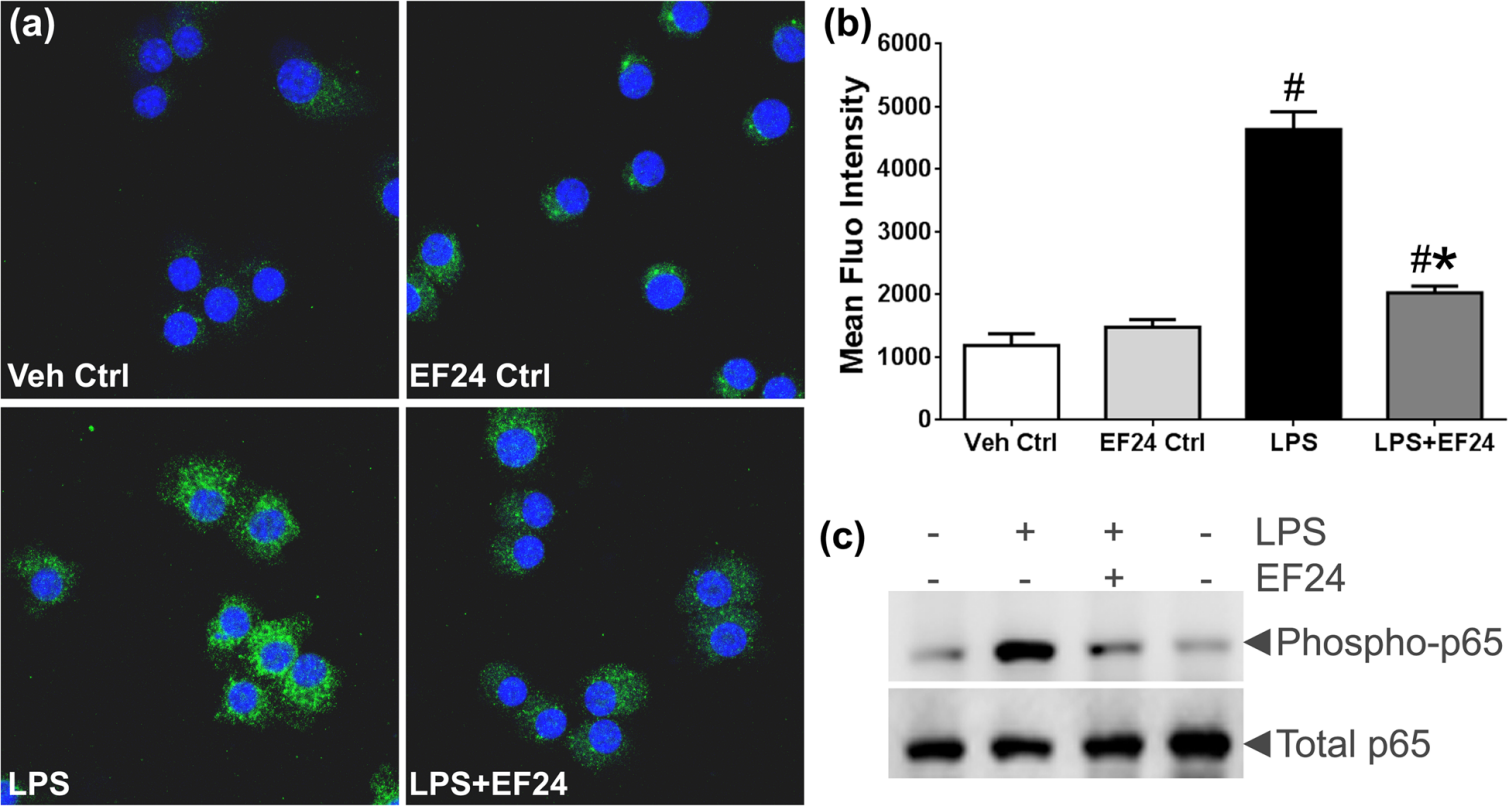
EF24 reduces expression of phospho-NF-kB p65 in LPS-stimulated DCs. JAWS II DCs were stimulated with LPS (100 ng/ml) for 4 h, followed 1 h by treatment with 10 μM of EF24. (**a**) For confocal microscopy (63X objective), the nucleus was stained with Hoechst dye (blue), whereas NF-kB was stained (green) with antibody against the phosphorylated form NF-kB p65. A representative set of images from three separate experiments is shown. (**b**) The intensity of green fluorescence representing phospho-NF-kB p65 (mean ± sem) was quantified in at least five different fields per view. One way analysis of variance and Bonferroni post-test was performed on the data (# *p* value < 0.05 vs. control and * *p* value < 0.05 vs. LPS treatment). (**c**) The expression of phospho-p65 and total p65 in JAWS II DCs, evaluated by immunoblotting

NF-kB is a known regulator of TLR4 and IL1-R1 expression. We monitored the activation of phospho-NF-kB after labeling the LPS-stimulated DCs with primary antibody against the phosphorylated form of p65 subunit of NF-kB (Fig. 4a). The confocal micrographs provided evidence that LPS induces the activation of NF-kB in DCs. EF24-treatment decreased the accumulation of phospho-p65 inside the LPS-stimulated DCs. The phospho-p65 MFI values in LPS-stimulated DCs increased to 4,689 ± 54 units from control levels of 1,290 ± 44 units (Fig. 4b). EF24 treatment significantly reduced the MFI value to 2,028 ± 21 units, though this value was significantly larger than the control levels. However, as compared to the EF24-control, the MFI value in LPS + EF24 group was not significantly different (Fig. 4b). These confocal results on phospho-p65 localization corroborate our earlier observations that EF24 reduces the DNA-binding and transcriptional activity of NF-kB in LPS-treated DCs [14]. We confirmed the effect of LPS and EF24 on phosphorylation of p65 by immunoblotting (Fig. 4c).

### EF24 does not affect the viability and growth characteristics of DCs

**Fig. 5:**
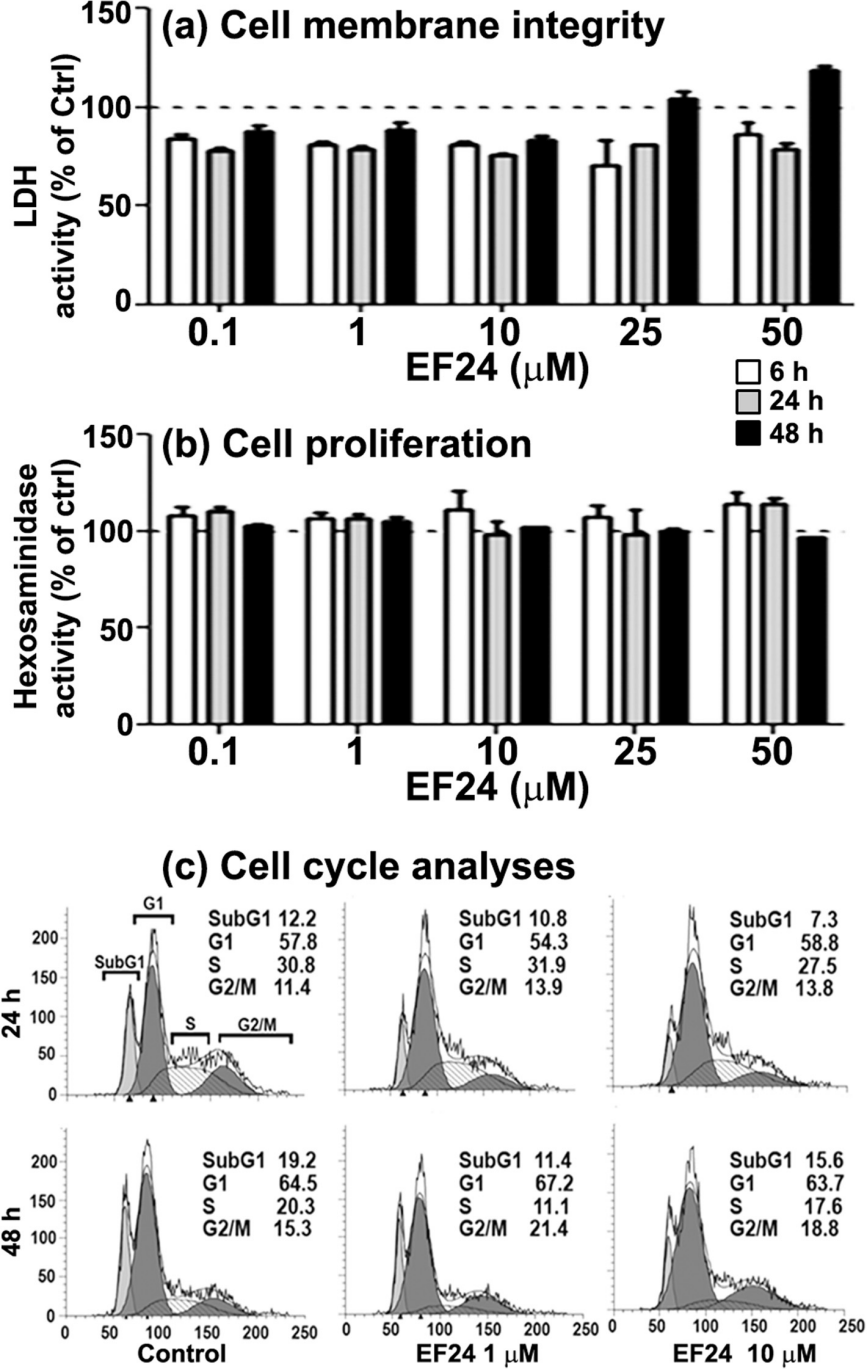
Effect of EF24 on the membrane integrity, proliferation, and cell cycle of JAWS II DCs. The cells were exposed to EF24 in the concentration range of 0.1-50 μM for up to 48 h. Membrane integrity was measured by (**a**) LDH activity in culture medium, whereas cell proliferation was quantified by (**b**) hexosaminidase enzyme assay. All the data (mean ± sem) are presented as percent of control (dotted line). (**c**) Flow cytometric analyses of cell cycle in JAWS II DCs treated with EF24 (1 and 10 μM). Propidium iodide staining was used to measure the DNA content of DCs after 24 and 48 h of EF24 exposure

Because of the widely known anti-proliferative effects of EF24 on cancer cells [27, 28, 20, 29], we examined the growth characteristics of EF24-treated DCs. The effect of EF24 on the viability of DCs was examined by monitoring the cell membrane integrity (LDH assay, Fig. 5a) and the proliferative capacity (hexosaminidase assay, Fig. 5b). We found that the membrane integrity and proliferation capacity of DCs remained unaffected after treatment with EF24 in the range of 0.1 - 50 μM. To further substantiate that EF24 does not affect normal cell growth characteristics of DCs, we performed cell cycle analysis of EF24-treated DCs. The histogram charts of propidium iodide-stained, vehicle-treated DCs are shown in Fig. 5c. Upon EF24 treatment up to 48 h, no major changes were observed in cells populating various stages of cell cycle. These observations imply that EF24 is not toxic to the DCs and does not adversely affect their growth pattern in the dose range used in this study.

## Discussion

In our recently published *in vivo* studies in a rat model of hemorrhagic shock, EF24 was found to act as a potent anti-inflammatory molecule for control of pulmonary inflammation [30]. The members of IL-1R superfamily constitute the most important pattern recognition receptors involved in this inflammatory process. The molecular signaling engendered by the interaction of DAMPs and other putative ligands with IL-1R members is the earliest response in the process [31]. Often, this early and protective response becomes a vicious and self-damaging inflammatory process, and provides the pathophysiological basis of multiple organ dysfunction syndrome [32]. As such, timely management of the inflammatory processes and participating molecules could prevent significant morbidity and mortality associated with inflammatory conditions such as trauma and shock. Herein, we report for the first time the effect of a chalcone derivative EF24 on the expression of IL-1R superfamily in LPS-stimulated DC model. The LPS-stimulated DCs or macrophages are the closest *in vitro* models for simulating the sterile inflammatory conditions observed in the *in vivo* scenarios of trauma and acute blood loss without infection. Unlike neutrophils and macrophages, DCs are also able to ‘preserve’ useful information about the antigen because of their low destructive capacity [33]. In other words, DCs are specialized to cleave exogenic protein to a level suitable for appropriate expression on class II MHC molecules, whereas neutrophils and macrophages do so less efficiently because of relatively higher destruction of antigenic protein sequence, often preceded by oxidative protein modification. We found that EF24 treatment suppressed the expression of pro-inflammatory IL-1R1 and TLR4, without affecting the expression of the negative regulators of inflammation, namely IL-1RA and IL-1R2. These effects of EF24 appear to be at transcriptional levels, perhaps mediated by NF-kB transcription factor. The observations are in line with our previous report, where EF24 potently inhibited NF-kB in an LPS-stimulated DCs model of sterile inflammation [14]. The effect of EF24 on NF-kB signaling has been reported to be dependent on its interaction and inhibition of IKKβ [19].

The two most important members of the IL-1R superfamily are TLR4 and IL-1R1. Although both TLR4 and IL-1R1 are placed upstream of NF-kB in the signaling network, their expression could be transcriptionally regulated by NF-kB. The NF-kB-mediated transcriptional control of TLR4 expression in response to LPS stimulation has been reviewed elsewhere [34, 35]. NF-kB recruits E2F1 as a transcription partner to activate LPS-responsive genes that include TLR4 mapped to 9q32-33 location in the chromosome [34]. In addition to the regulation at transcriptional level, NF-kB also influences TLR4 expression by post-transcriptional mechanisms. For instance, TLR4 mRNA is stabilized by an RNA-binding protein called human antigen R (HuR) [35–37]. HuR is a direct transcriptional target of NF-kB [38]. Although it is not clear at present whether the effect of NF-kB inhibition on HuR is involved, a recent study has described that GL63 (a COX-2 inhibiting curcumin analog which closely resembles EF24) reduces the cytoplasmic localization and protein abundance of HuR in lung cancer H460 cells [39].

Given these reports, it could be speculated that the reduced TLR4 expression in our model of LPS-stimulated DCs is because of EF24-mediated inhibition of NF-kB. Interestingly, EF24 pre-treatment also suppressed LPS-induced expression of TLR4 (Fig. 1b). This observation is significant in suggesting that EF24 could serve as prophylactic as well as therapeutic agent in inflammatory conditions. It could be explained by the ability of EF24 to inhibit IKK activation by physically interacting with its cysteinyl groups [16]. Besides phosphorylation of IkBα, IKK has been also shown to phosphorylate RelA/p65 in LPS-stimulated macrophages [40]. Thus, the inability of IKK-EF24 complex to serve as a kinase inhibits the induction of canonical NF-kB pathway by LPS stimulation.

NF-kB also controls the expression of IL-1R1, but by mechanism different from that for TLR4 expression. The pro-inflammatory signaling of NF-kB pathway results in upregulation of synthesis and secretion of IL-1 and IL-6 [41, 42]. The murine IL-1α and IL-1β are mapped close to human region 2q12, but murine IL-1R1 is mapped to chromosome 1 in a region of synteny with human chromosome 2 [43]. We show in Fig. 4 that EF24 treatment of DCs reduces nuclear translocation of p65 subunit of NF-kB. The nuclear translocation of activated NF-kB is dependent on phosphorylation of p65 subunit (Rel A). Therefore, nuclear expression of phosopho-p65 subunit of NF-kB is a marker of NF-kB activation. Activated NF-kB binds to the promoter regions of pro-inflammatory genes and upregulates the transcription of pro-inflammatory cytokines [44]. Proinflammatory IL-1β, which was induced by LPS treatment of DCs (Fig. 3c), is also regulated by NF-kB-mediated transcriptional control [45]. NF-kB inhibition results in reduced levels of these cytokines, which in turn attenuate the signaling through IL-1R1. It has been previously shown by us that EF24 treatment of LPS-stimulated DCs reduces the production of pro-inflammatory cytokines [14].

Like in the case of steroids, the suppression of IL-1R members and reduced content of pro-inflammatory mediators could raise a valid question about the undesirable immunosuppressive side-effects of EF24. In fact much of the anti-inflammatory effects of steroidal drugs are associated with functional suppression of the beneficial aspects of immune response [46, 47]. More recently, Patterson et al reported that fluticasone inhalation impairs the pulmonary clearance of *Klebsiella pneumoniae* in a mouse model [47].

However, this concern in the case of EF24 is addressed by our observation that EF24 does not affect the phagocytic bacterial uptake or localization in the DCs [48]. Phagocytosis is a highly conserved primary innate mechanism employed by antigen presenting cells to ward off infection from the body, and the phagocytic capture and presentation of antigen is a key feature of functional DCs [33].

Whereas preservation of phagocytic function of antigen presenting cells is desirable, it is also required to assess the processes contributing in antigen presentation by DCs. Following phagocytosis, the antigen presentation phenomenon in DCs employs another important intracellular process called autophagy. Autophagic process enables the delivery of cytoplasmic material to the lysosome and presentation of antigen by MHC class II molecules [49, 50]. In our previous report we showed that EF24 reduced the expression of LPS-induced MHC class II, CD80 and CD86 molecules, and abrogated the appearance of dendrites in DCs [14]. These molecules play a critical role in antigen presentation which is preceded by a conserved process of autophagy whose role in DC-mediated T cell priming, antigen presentation, and cytokine production has been demonstrated [51, 52]. By monitoring the expression of LC3B expression, we found that EF24 treatment maintained the basal autophagy process, but reduced LPS-stimulated autophagy (data not shown). Exaggerated induction of autophagy in DCs after LPS treatment could be counterproductive because of the extensive degradation of antigenic information. Therefore, the suppressive effect of EF24 on LPS-induced autophagy may be beneficial in preserving antigen information.

## Conclusions

The results discussed in this report indicate that EF24 reduces the expression of pro-inflammatory IL-1R1 and TLR4. Given the purported mechanism of action of EF24, this effect is speculated to be dependent on NF-kB inhibition. Since the discovery of EF24 [27] and its subsequent testing has revolved around its potent anti-proliferative activity in cancer cells [28, 20, 29], a potential concern about the utility of EF24 as an anti-inflammatory agent exists because of its pro-apoptotic effect. However, we found no effect of EF24 on the viability and cell cycle of DCs. Here, it must be noted that these experiments were carried out in immortalized DCs, and toxicity of EF24 in primary DCs, such as those derived from bone marrow, must also be evaluated. These *in vitro* observations are suggestive of the potential utility of EF24 in control of hemorrhage-induced inflammation because drugs modulating transcriptional control of inflammatory processes are suggested to be more effective than steroids and selective COX-2 inhibitors [53]. We have recently reported that EF24 is remarkably effective in suppressing pulmonary inflammation in a rat model of hemorrhagic shock [30].
